# Effects of bone-specific physical activity on body composition, bone mineral density, and health-related physical fitness in middle-aged women

**DOI:** 10.20463/jenb.2019.0030

**Published:** 2019-12-31

**Authors:** Sung-Woo Kim, Sung-Woo Jung, Myong-Won Seo, Hun-Young Park, Jong-Kook Song

**Affiliations:** 1 Physical Activity and Performance Institute (PAPI), Konkuk University, Seoul Republic of Korea; 2 Department of Taekwondo, College of Physical Education, Kyung Hee University, Yongin Republic of Korea; 3 Department of Sports Medicine and Science, Konkuk University, Seoul Republic of Korea

**Keywords:** bone-specific physical activity, body composition, bone mineral density, health-related physical fitness, middle-aged women

## Abstract

**[Purpose]:**

The study aimed to determine the effects of bone-specific physical activity on body composition, bone mineral density (BMD), and health-related physical fitness in middle-aged women.

**[Methods]:**

One hundred eighty-six middle-aged women aged 31-49 years participated in this study. The subjects were divided into tertile groups according to the level of physical activity (low-score group, n=62; middle-score group, n=62; high-score group, n=62). Bone-specific physical activity participation was assessed using the bone-specific physical activity questionnaire. Body composition and BMD were measured using dual-energy X-ray absorptiometry. Health-related physical fitness test included isometric muscle strength (grip strength), muscular endurance (sit-ups), flexibility (sit and reach), and cardiorespiratory fitness (maximal oxygen uptake [VO2max]).

**[Results]:**

The high-score group had a significantly higher fat-free mass (*p*=.045, partial eta-squared value [η_p_^2^]=.033) than the middle- and low-score groups, whereas the high-score group had significantly lower percent body fat (*p*=.005, η_p_^2^=.056) than the other two groups. Whole-body BMD (*p*=.034, η_p_^2^=.036) and lumbar BMD (*p*=.003, η_p_^2^=.060) were significantly higher in the high-score group than in the low-score group. The high-score group performed significantly better for grip strength (*p*=.0001, η_p_^2^=.101), sit-ups (*p*=.0001, η_p_^2^=.108), and VO2max (*p*=.0001, η_p_^2^=.092) than the other two groups.

**[Conclusion]:**

The present study suggests that bone-specific physical activity could be useful in improving body composition, BMD, and health-related physical fitness in middle-aged women, significantly enhancing their BMD and health conditions.

## INTRODUCTION

The Lancet Physical Activity Series Working Group reported that approximately 31% of adults worldwide have insufficient physical activity (PA), resulting in several chronic diseases, such as stroke, osteoporosis, diabetes, and hypertension, which are the major risk factors for mortality^1,2^. Moreover, insufficient PA has a negative impact on cardiovascular diseases, including coronary artery disease and stroke^3-5^. Lozano et al.^6^ reported that cardiovascular deaths accounted for 29.6% of all-cause mortality, which was two times higher than that caused by cancer. Individuals who participate in regular physical activities have a lower risk of being diagnosed with cardiovascular diseases than individuals who are engaged in a sedentary lifestyle^7,8^. It was also revealed that premature mortality could be reduced to approximately 20% when individuals participate in PA regularly^9^. Unfortunately, 85% of adults (men and women) do not regularly participate in moderate-to-vigorous PA greater than 150 min per week, and the incidence of being diagnosed with a disease, as a result of insufficient PA, did not decrease over the last 10 years^10-12^. Furthermore, the incidence of metabolic diseases, such as obesity, cardiovascular disease, and type 2 diabetes, is increasing in adults due to insufficient PA^13,14^. Therefore, middle-aged women need to participate in regular PA because it can not only reduce the risk of disease but also improve health and manage PA levels according to their life cycle.

Body composition consistently changes throughout life, which is caused by various factors such as aging, dietary intake, and PA. Generally, the amount of fat increases but muscle mass and bone mineral density (BMD) decrease with aging^15,16^. Particularly, muscle mass gradually decreases from the late 20s with a ratio of 0.4-0.8 kg per decade^17^. Moreover, premenopausal women’s BMD is associated with weight, while it is less associated with fat and more dependent on fat-free mass (FFM)^18^. For women, after reaching 90% of the peak bone mass at age 18, BMD begins to decrease at a relatively slow rate and rapidly decreases at a rate of 0.5%-1% per year after menopause^19-21^. Therefore, increasing the peak bone mass and maintaining BMD during adulthood are significantly important. Middle-aged women participate in regular physical activities to maintain high BMDs and minimize bone loss, reducing the risk of fractures and osteoporosis that can occur after menopause.

PA and physical fitness are important factors in promoting health status^13^. According to the previous studies^22-24^, health-related physical health can be affected not only by lifestyle and genetic factors but also by participation in regular PA. Moreover, aging and insufficient PA will lead to a decrease in health-related physical fitness and daily functional impairment^25,26^. The maximal oxygen uptake (VO2max), which is an index of cardiovascular endurance in the health-related physical fitness, is reduced by approximately 3%-6% with aging^27^. High levels of cardiopulmonary endurance and muscle mass maintained from adulthood can reduce the incidence of cardiovascular and metabolic diseases such as hypertension and diabetes^28,29^. Additionally, physical fitness, which is achieved through regular PA, also exhibits a positive impact on life expectancy by reducing mortality from cardiovascular diseases^30^. Thus, health-related physical fitness is an indirect health indicator of the body, and it is significantly necessary to manage it continuously by participating in regular PA.

The PA research tools and methods used in the previous studies did not examine the type of PA that may impact bone health. However, the bone-specific physical activity questionnaire (BPAQ), which considers the type of PA that could directly impact bone health, has recently been developed and used in some previous studies^31-35^. Therefore, our study aimed to determine the effects of bone-specific PA on body composition, BMD, and health-related physical fitness in middle-aged women.

## METHODS

### Participants

A total of 205 middle-aged women aged 31-49 years were randomly selected. Based on their past medical history, these women had no medical problems. However, 19 participants were excluded due to personal reasons. Overall, this study included 186 middle-aged women. According to the PA level estimated using the BPAQ, participants were divided into tertile groups (low-score group, n=62; middle-score group, n=62; high-score group, n=62)^36^. The characteristics of the participants are shown in Table 1.

**Table 1.** Physical characteristics and bone-specific physical activity questionnaire scores by physical activity level

	Low-score group (n=62)	Middle-score group (n=62)	High-score group (n=62)	*F*-value	Prob > *F*
Age (yrs)	40.4±4.06a	41.1±4.13a	39.8±3.94a	1.525	.220
Body height (cm)	161.1±4.85a	160.7±5.24a	162.7±5.08a	2.802	.063
Body weight (kg)	58.8±8.59a	58.9±9.88a	59.0±9.02a	.014	.986
BMI (kg/m^2^)	22.6±3.14a	22.8±3.61a	22.3±3.10a	.429	.652
Current BPAQ score	0.3±0.46a	1.9±3.12a	5.8±13.93b	7.368	.001
Past BPAQ score	1.7±0.79a	8.7±5.39b	37.0±20.31c	146.784	.0001
Total BPAQ score	1.0±0.44a	5.3±2.49b	21.4±10.66c	179.732	.0001

Note. Values are expressed as mean ± standard deviation.

Different lowercase letters indicate significant differences among the groups

BMI, body mass index; BPAQ, bone-specific physical activity questionnaire

### Study design

The participants from Gyeonggi-do Province were recruited from January to February 2018, and they were randomly sampled through posters and flyers. Before the study, the objectives, procedures, advantages, and potential disadvantages of this study were explained to all participants. This study was approved by the Institutional Review Board of Kyung Hee University (KHSIRB-17-048). Each participant provided written informed consent. On the testing day, body composition (FFM, fat mass [FM], and percent body fat), BMD (whole-body, lumbar, femur, and forearm region), health-related physical fitness (grip strength [GS], sit-ups [SU], sit and reach [SAR], and VO2max), and BPAQ (current BPAQ [cBPAQ], past BPAQ [pBPAQ], and total BPAQ [tBPAQ]) were measured between 9:00 and 11:00 AM.

### Measurements

**Body composition and bone mineral density**

The body height and weight were measured, and body mass index was calculated by dividing the body weight (kg) by the square of body height (m^2^). Body composition and BMD were measured using dual-energy X-ray absorptiometry, with a Hologic QDR 4500W bone densitometer (Hologic, Marlborough, USA). Body compositions, including FM, percent body fat, and FFM, were analyzed. All the participants were scanned at four different sites of the BMD (e.g., whole-body, lumbar, femur, and forearm region).

**Health-related physical fitness**

Health-related physical fitness test included isometric muscle strength (GS), muscular endurance (SU), flexibility (SAR), and cardiorespiratory fitness (VO2max). The graded exercise stress test (GXT) using the Bruce protocol was performed to measure the VO2max. Oxygen uptake was measured using a Quark b2 (COSMED, Rome, Italy). All participants started walking at 1.7 mph with a 10% gradient. The speed was increased to 0.8-0.9 mph at 3-min intervals, and the incline was increased by 2% with each stage. The GXT was performed on a treadmill (Series 2000, Marquette Electronics, Wisconsin, USA). Maximal heart rate (HRmax) was measured using a heart rate monitor (Polar RS400, Polar Electro Oy, Kempele, Finland). VO2max was defined as follows. The participants should meet three out of the following four criteria: (1) oxygen uptake alteration should not exceed 2.1 ml/kg/min (VO_2_ plateau), (2) heart rate should not increase regardless if the stage was increased, (3) the ratings of perceived exertion (RPE) should be >17 (RPE of 6-20), and (4) RER should be over 1.1^37^.

**Bone-specific physical activity questionnaire**

The BPAQ assessment instrument aims to evaluate the PA level that exerts a mechanical load on the bone^31^. Participants were asked to complete the two independent sections of the BPAQ, that is, the pBPAQ (previous PA level from birth to 12 months) and the cBPAQ (previous 12-month period PA level). The online Microsoft Visual Basic program was used to generate the pBPAQ, cBPAQ, and tBPAQ scores.

### Statistical analysis

The power test was performed using G*Power 3.1.9.2 (Franz Faul, University of Kiel, Kiel, Germany) with an effect size of 0.25, a significance level of 0.05 (α=0.05), and a power of 0.8 for all statistical tests. G*Power showed that 159 participants had sufficient power for this study.

Statistical analyses were performed using the Statistical Analysis System (SAS) software version 9.4 (SAS Institute, Cary, NC, USA). The mean, standard deviation, and 95% confidence interval were calculated. One-way analysis of variance was used to determine the differences among the three groups on the dependent variables and was followed by Duncan’s post-hoc multiple range test. The effect size was computed as partial eta-squared values (η_p_^2^; small, ≥.01; medium, ≥.06; large, ≥.14). The statistical significance level was set at 0.05.

## RESULTS

### Body composition

Table 2 indicates the difference of body composition in middle-aged women by bone-specific PA level. There were no significant differences in FM (*F*=1.566, *p*=.212, η_p_^2^ =.017) among the three groups. However, significant differences were observed on FFM (*F*=3.153, *p*=.045, η_p_^2^=.033) and percent body fat (*F*=5.427, *p*=.005, η_p_^2^=.056) among the three groups. The high-score group (38.8±4.61 kg) had significantly higher FFM than the middle- (36.9±4.92 kg) and low-score groups (37.0±4.07 kg). On the contrary, the high-score group (28.9±6.39%) had significantly lower percent body fat than the other two groups (middle-score group, 32.0±5.89%; low-score group, 31.9±5.43%).

**Table 2.** Comparison of body composition among the three groups

	Low-score group (95% CI)	Middle-score group (95% CI)	High-score group (95% CI)	*F*-value	Prob > *F*	η_p_^2^
Fat mass (kg)	18.8±5.56a (17.3-20.2)	18.8±6.02a (17.4-20.4)	17.2±5.94a (15.8-18.8)	1.566	.212	.017
Fat-free mass (kg)	37.0±4.07a (36.0-38.2)	36.9±4.92a (35.8-38.2)	38.8±4.61b (37.6-39.9)	3.153	.045	.033
Percent body fat (%)	31.9±5.43a (30.5-33.3)	32.0±5.89a (30.6-33.4)	28.9±6.39b (27.4-30.7)	5.427	.005	.056

Note. Values are expressed as mean ± standard deviation.

Different lowercase letters indicate significant differences among the groups

95% CI, 95% confidence interval

### Bone mineral density

Table 3 shows the significant differences in whole-body BMD (*F*=3.446, *p*=.034, η_p_^2^=.036) and lumbar BMD (*F*=5.881, *p*=.003, η_p_^2^=.060) among the three groups. However, significant differences were not observed for femur BMD (*F*=1.609, *p*=.203, η_p_^2^=.017) and forearm BMD (*F*=.053, *p*=.949, η_p_^2^=.001) among the three groups. The high-score group (1.148±0.087 g/cm^2^) had significantly higher whole-body BMD than the low-score group (1.113±0.070 g/cm^2^). Moreover, the high-score group (1.057±0.114 g/cm^2^) had significantly higher lumbar BMD than both the middle- (1.004±0.106 g/cm^2^) and low-score groups (0.993±0.109 g/cm^2^).

**Table 3.** Comparison of bone mineral density among the three groups

	Low-score group (95% CI)	Middle-score group (95% CI)	High-score group (95% CI)	*F*-value	Prob > *F*	η_p_^2^
Whole-body BMD (g/cm^2^)	1.113±0.070a (1.096-1.132)	1.121±0.072ab (1.101-1.139)	1.148±0.087b (1.128-1.170)	3.446	.034	.036
Femur BMD (g/cm^2^)	0.864±0.104a (0.840-0.890)	0.860±0.100a (0.835-0.885)	0.890±0.098a (0.867-0.915)	1.609	.203	.017
Lumbar BMD (g/cm^2^)	0.993±0.109a (0.965-1.022)	1.004±0.106a (0.978-1.033)	1.057±0.114b (1.030-1.086)	5.881	.003	.060
Forearm BMD (g/cm^2^)	0.562±0.036a (0.553-0.571)	0.561±0.032a (0.553-0.569)	0.563±0.034a (0.554-0.572)	.053	.949	.001

Note. Values are expressed as mean ± standard deviation.

Different lowercase letters indicate significant differences among the groups

95% CI, 95% confidence interval; BMD, bone mineral density

### Health-related physical fitness

Table 4 indicates the insignificant differences in SAR (*F*=1.960, *p*=.114, η_p_^2^=.021) and HRmax (*F*=.718, *p*=.489, η_p_^2^=.008) among the three groups. However, significant differences were observed for GS (*F*=10.334, *p*=.0001, η_p_^2^=.101), SU (*F*=11.059, *p*=.0001, η_p_^2^=.108), and VO2max (*F*=9.278, *p*=.0001, η_p_^2^=.092) among the three groups. The high-score group had significantly higher GS (29.2±4.16 kg), SU (21.3±9.21 n), and VO2max (34.8±7.11 ml·kg^-1^·min^-1^) than the middle- (GS, 26.0±4.30 kg; SU, 16.4±10.27 n; VO2max, 31.1±4.86 ml·kg^-1^·min^-1^) and low-score groups (GS, 26.5±4.28 kg; SU, 13.2±9.43 n; VO2max, 30.9±4.84 ml·kg^-1^·min^-1^).

**Table 4.** Comparison of health-related physical fitness among the three groups

	Low-score group (95% CI)	Middle-score group (95% CI)	High-score group (95% CI)	*F*-value	Prob > *F*	η_p_^2^
Sit and reach (cm)	10.2±9.43a (7.7-12.5)	10.5±9.20a (8.3-12.8)	13.4±10.81a (10.5-15.9)	1.960	.144	.021
Grip strength (kg)	26.5±4.28a (25.5-27.6)	26.0±4.30a (24.9-27.0)	29.2±4.16b (28.2-30.2)	10.334	.0001	.101
Sit-ups (n)	13.2±9.43a (10.9-15.7)	16.4±10.27a (13.9-18.9)	21.3±9.21b (18.9-23.6)	11.059	.0001	.108
VO2max (ml·kg^-1^·min^-1^)	30.9±4.84a (29.7-32.2)	31.1±4.86a (29.8-32.3)	34.8±7.11b (32.9-36.6)	9.278	.0001	.092
HRmax (beats·min^-1^)	173.5±10.64a (170.9-175.8)	172.5±8.24a (170.2-174.5)	174.6±10.33a (172.0-177.5)	.718	0.489	.008

Note. Values are expressed as mean ± standard deviation.

Different lowercase letters indicate significant differences among the groups

95% CI, 95% confidence interval; VO2max, maximal oxygen uptake; HRmax, maximal heart rate

## DISCUSSION

The present study examined the effects of bone-specific PA on body composition, BMD, and health-related physical fitness in middle-aged women. This study revealed that bone-specific PA reduced percent body fat and increased FFM, whole-body BMD, lumbar BMD, GS, SU, and VO-2max.

It is well known that regular PA increases FFM and decreases percent body fat^38^. The results of this study showed that FFM in the high-score group was significantly higher than those in the middle- and low-score groups. Additionally, the high-score group showed a significantly lower percent body fat compared to the other two groups. These results are consistent with the results of the previous studies. Donnelly et al.^39^ reported that there was no difference in FM between women with a high PA level and a low PA level. Moreover, Thompson et al.^40^ reported that percent body fat in the high PA group was significantly lower than that in the low PA group in middle-aged women when measured by PA levels using the pedometer. Saravi and Sayegh^18^ reported that FFM in the habitual PA group was significantly higher than that in the sedentary group in premenopausal women when measured by PA levels using the IPAQ. Generally, participation in regular PA increases FFM and decreases percent body fat in middle-aged premenopausal women, which helps in preventing obesity and sarcopenia. Therefore, regular PA is highly recommended to improve body composition.

A lifestyle change (e.g., regular participation in PA, nutrition intake, and cessation of smoking and alcohol intake) is an essential goal to prevent osteoporosis and fractures. The results of BMD in our study showed that whole-body BMD in the high-score group was significantly higher than that in the low-score group. Moreover, the high-score group showed significantly higher lumbar BMD compared to the other two groups. These results were consistent with the results of the previous studies, which confirmed that the high PA level had a positive impact on femur BMD and lumbar BMD^32,36,41^. Furthermore, it was stated that healthy young adults with a high PA level resulted in higher lumbar BMD and higher femur BMD^42^. Morseth et al.^43^ reported that a high PA level was closely associated with high femoral BMD in healthy female adults. Additionally, the comparison of BMD based on the PA level in middle-aged women showed that women with a high PA level had higher lumbar BMD, femur BMD, and whole-body BMD than women with a low PA level^44-46^. Ultimately, it was revealed that women who have a high PA level during adulthood had a low risk of osteoporosis during late adulthood^43,47^. Therefore, to prevent osteopenia and osteoporosis and to restrain the rate of decrease in BMD with aging, it is necessary to participate in various physical activities, including high-impact exercise.

With aging, women’s musculoskeletal fitness and cardiorespiratory endurance gradually decrease^48^. However, participating in regular PA could improve musculoskeletal fitness, reducing the risk of chronic diseases and enhancing the overall health conditions^49^. The results of health-related physical fitness in this study showed that GS, SU, and VO2max in the high-score group were significantly higher than those in the middle- and low-score groups. Based on a previous study, female adults with a high PA level showed higher VO2max than female adults with a low PA level, and high cardiovascular endurance was positively associated with a high PA level^50^. Moreover, it was confirmed that the high PA level is positively associated with high physical fitness, which showed a beneficial effect on health conditions^48^. Therefore, participating in regular PA plays a vital role in improving health-related physical fitness and helps prevent musculoskeletal and cardiovascular diseases.

The present study revealed that a bone-specific PA reduced percent body fat and increased FFM, whole-body BMD, lumbar BMD, GS, SU, and VO2max in middle-aged women. We believe that a bone-specific PA could be useful in improving body composition, BMD, and health-related physical fitness in middle-aged women, ultimately enhancing their BMD and health conditions.
